# Orthopœdic Institution—Prof. Mott

**Published:** 1841-09

**Authors:** 


### ORTHOPOEDIC INSTITUTION—PROFESSOR MOTT.

The profession will be glad to learn that Professor Mott of New York, well known as one of the most distinguished surgeons of the age, is about to open an institution for the treatment of club-foot, spinal curvatures, and other analogous distortions of the human frame. It is located at the village of Bloomingdale, on the banks of the Hudson river, six miles from New York, and is to be conducted on the same plan as that now in such successful operation under the direction of M. Julius Guèrin, the distinguished orthopœdic surgeon in Paris, and several similar establishments on the continent of Europe. During his residence in the old world, embracing a period of six years, Dr. Mott paid particular attention to this class of deformities, and is therefore in possession, not only of ample experience, but also of the requisite apparatus. Such an institution has been long needed, and we need scarcely add that we wish its distinguished founder every possible success. G.

